# Supplementary material for: GPCR Binding and JNK3 Activation by Arrestin-3 Have Different Structural Requirements
Source: Cells. 2023 Jun 6;12(12):1563. doi: 10.3390/cells12121563 (PMC10296906; doi:10.3390/cells12121563)
Supplement: Supplementary file 1 [file cells-12-01563-s001.zip › cells-2403495-supplementary.pdf]

GPCR binding and arrestin-3-dependent JNK3 activation have different structural requirements

Chen Zheng[a], Liana D. Weinstein[a], Kevin K. Nguyen[a], Abhijeet Grewal[a], Eugenia V. Gurevich[a], Vsevolod V. Gurevich[a*]

Supplementary Information

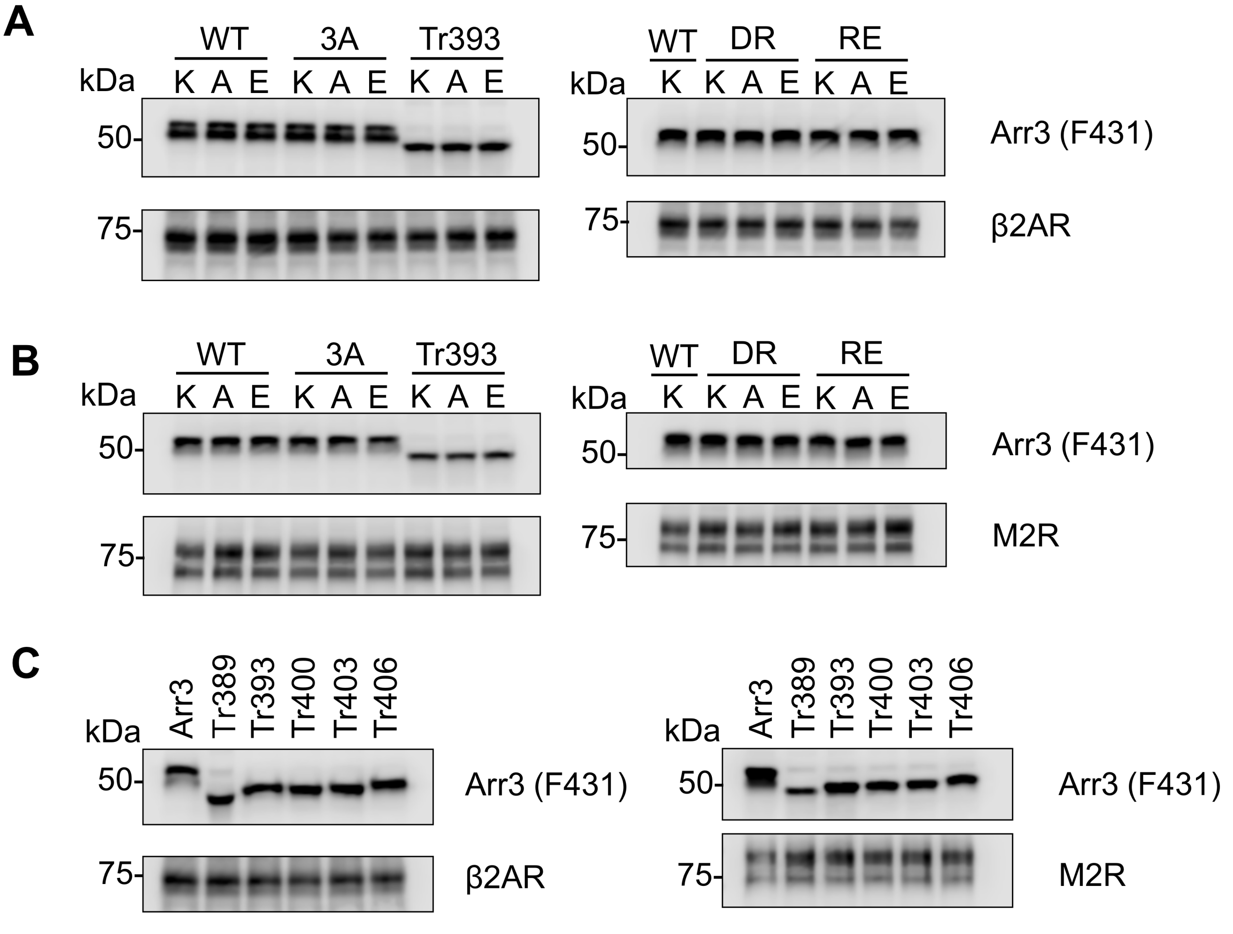

Fig. S1. **Expression of SmBiT-arrestin-3 and LgBiT-GPCRs used in nanoBiT assay.** Arrestin-3 and receptors were detected by western blot with anti-arrestin F431 [1] and anti-HA (#3724, Cell Signaling Technology) antibodies, respectively. **A.** Samples shown in Fig. S3. **B.** Samples shown in Fig. S4. **C.** Samples shown in Fig. 3.

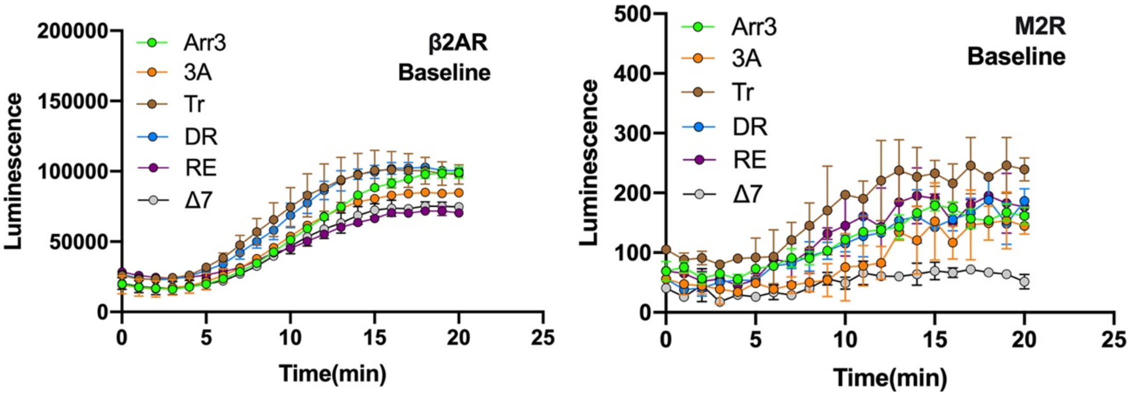

Fig. S2. **Basal luminescence (before agonist stimulation) in cells expressing LgBiT-tagged β2AR and M2R with SmBiT-tagged WT arrestin-3 and indicated mutants shown in Fig. 2.** Note that the signal with β2AR is much larger than with M2R. The most likely explanation is that β2AR has phosphorylation sites in the C-terminus, where LgBiT is fused, which binds in the cavity of the N-domain [2], close to the SmBiT fused to the N-terminus of arrestin-3. In contrast, M2R has phosphorylation sites necessary for arrestin binding in the third cytoplasmic loop [3,4], while the LgBiT is fused to its short C-terminus that does not have any phosphorylation sites.

A

Venus

Venus-Arr3

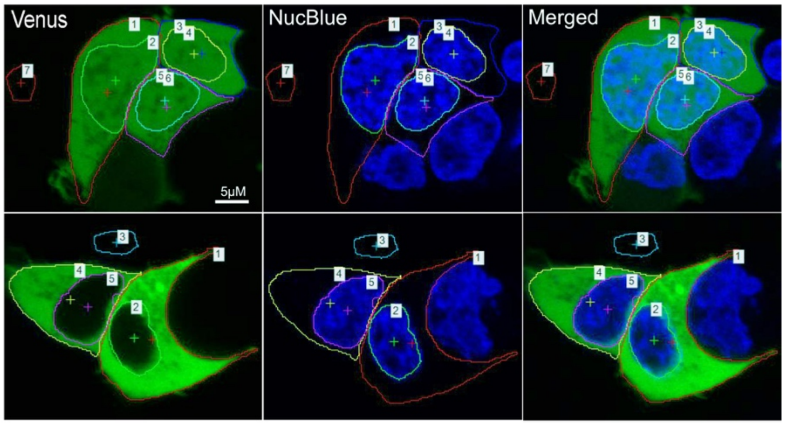

B

Venus				Green Fluorescence (488 nm)		NET Fluorescence		Normalization	
Cell#	Area#	Field	ROI, pixels	MEAN Intensity per pixel	Total Intensity	MEAN Intensity per pixel	Total Intensity	MEAN Nuclear Intensity per px/MEAN Total/px	Nuclear content, % of Total)
1	1	Total	7506	75.04	563213	73.56	552104	1.0150	49%
	2	Nuclear	3592	76.15	273535	74.67	268219		
2	3	Total	3772	83.52	315030	82.04	309447	0.9950	51%
	4	Nuclear	1935	83.07	160734	81.59	157870		
3	5	Total	3797	77.29	293461	75.81	287841	1.0450	57%
	6	Nuclear	2080	80.72	167904	79.24	164826		
Background	7	Background	627	1.48	930	-	-		

Venus-Arr3				Green(GFP)		NET Fluorescence		Normalization	
Cell#	Area#	Field	ROI	MEAN Intensity per pixel	Total Intensity	MEAN Intensity per pixel	Total Intensity	MEAN Nuclear Intensity per px/MEAN Total/px	Nuclear content, % of Total
1	1	Total	5229	125.31	655270	123.83	647531	0.3520	10%
	2	Nuclear	1531	45.07	69005	43.59	66739		
2	4	Total	3980	67.49	268630	66.01	262740	0.3730	18%
	5	Nuclear	1890	26.12	49365	24.64	46568		
Background	3	Background	512	1.01	518	-	-		

Fig. S3. Quantification of the subcellular distribution of wild type and mutant arrestin-3. HEK293 arrestin-2/3 KO cells were co-transfected with HA-ASK1, HA-JNK3α2 and either control (Venus) or indicated N-terminally Venus-tagged forms of arrestin-3 (to mimic the condition of the JNK activation). The images were collected from live cells 48 h post-transfection on the Olympus confocal microscope, as described in Methods. The images were analyzed for the intensity of the green (488 nm) fluorescence using NIS-elements software. **(A)** Representative cells expressing Venus or Venus-tagged WT arrestin-3 (Venus-Arr3). The total cell area and the nuclear area (stained by NucBlue) are outlined representing Regions of Interest (ROI) marked by numbers for the analysis. A cell-free area is used to determine the background (#7 in Venus and #3 in Venus-Arr3). **(B)** The software provides measurements of ROI in pixels, fluorescence intensity per pixel and sum of intensity for the entire ROI. NET values are measurements after the

subtraction of the background. To estimate a relative enrichment of the arrestin-3 protein in the nucleus, we used the mean nuclear fluorescence intensity per pixel normalized to the mean total intensity per pixel (to account for the differences in the levels of transfection among the cells). To estimate the overall distribution of the arrestin-3 proteins between the nucleus and the cytosol, we calculated the percentage of the total expressed protein residing in the nucleus (nuclear fluorescence as % of the total fluorescence).

1. Vishnivetskiy, S.A., Zhan, X., Chen, Q., Iverson, T.M., and Gurevich, V.V. (2014). Arrestin expression in E. coli and purification. Curr Protoc Pharmacol *67*, Unit 2.11.11-19.
2. Lee, Y., Warne, T., Nehmé, R., Pandey, S., Dwivedi-Agnihotri, H., Chaturvedi, M., Edwards, P.C., García-Nafría, J., Leslie, A.G.W., Shukla, A.K., and Tate, C.G. (2020). Molecular basis of β-arrestin coupling to formoterol-bound β(1)-adrenoceptor. Nature *583*, 862-866. 10.1038/s41586-020-2419-1.
3. Nakata, H., Kameyama, K., Haga, K., and Haga, T. (1994). Location of agonist-dependent-phosphorylation sites in the third intracellular loop of muscarinic acetylcholine receptors (m2 subtype). Eur J Biochem *220*, 29-36.
4. Lee, K.B., Ptasienski, J.A., Pals-Rylaarsdam, R., Gurevich, V.V., and Hosey, M.M. (2000). Arrestin binding to the M2 muscarinic acetylcholine receptor is precluded by an inhibitory element in the third intracellular loop of the receptor. J Biol Chem *275*, 9284-9289.
